# Where the DNA hides... In praise of José Rodrigues Coura

**DOI:** 10.1590/0074-02760220146

**Published:** 2022-07-15

**Authors:** Adeilton Alves Brandão

**Affiliations:** 1 Fundação Oswaldo Cruz-Fiocruz, Instituto Oswaldo Cruz, Rio de Janeiro, RJ, Brasil

In the biomedical sciences timeline, 2021 will be marked by two major events: (a) the second year of Covid-19 pandemics and its huge impact on our society; (b) the consolidation of new vaccine technology to deal with emergent infectious agents. For the Brazilian community of researchers in tropical medicine nonetheless, it is also a year that will be remembered as the farewell to José Rodrigues Coura (1927-1921), medical doctor, professor and researcher in human infectious diseases, director of the Instituto Oswaldo Cruz (IOC), vice president of research at Fiocruz, author of hundreds of scientific articles, editor and enthusiastic collaborator of the journal Memórias do Instituto Oswaldo Cruz (MIOC). The relevance of José Rodrigues Coura for MIOC can be realised from one particular historical event: from 1977 to 1979 the journal did not publish a single article! Prof. Coura, soon after starting his job as director of IOC, resumed the publishing operation of MIOC and afforded it the current scope as an international journal dedicated to publish research work in human infectious diseases, their agents and vectors.

I was introduced to Prof. Coura in the beginning of year 2000 when I was hired as a post-doctoral research fellow of the now extinct department of Tropical Medicine of the IOC. During our brief conversation Prof. Coura told me: “So, you are the person who will show us where the DNA hides…!” I immediately thought: “this honorable Professor is joking! Every sophomore knows that DNA is not hidden!”. As it happens to most of the young scientists trained in molecular biology, I was convinced that the molecular toolbox was powerful enough to solve every problem in biology if we could reduce it to its constituent blocks of nucleic acid and proteins. With respect to DNA of protozoa parasites, *e.g.*, *Trypanosoma cruzi*, *Leishmania* sp., *Plasmodium* sp. there was no secret to its exact location. What was really unknown to me at that moment was a peculiar trace of Coura’s personality: the fine sense of humor coupled to sarcastic hints about the tasks the researchers of tropical medicine should embrace. In my case, I was starting a collaboration with the research group leaded by Octavio Fernandes, a brilliant young researcher in infectious diseases, who had few years earlier gotten a permanent position in Coura’s lab and was in pursuit of novel DNA based molecular markers to better describe/identify the wide range of *T. cruzi* and *Leishmania* sp strains.

That was the beginning of an intense “living together” in the Arthur Neiva heritage building, home of the Tropical Medicine department until 2007, and from then on for the three laboratories that remained after the department extinction. Only one out of these laboratories was really new, the Laboratory for Molecular Epidemiology of Infectious Diseases (LEMDI, also extincted), which was created by Octavio Fernandes as a spin off from the Coura´s laboratory, the extant Laboratory of Parasite Diseases. At that time, the environment in the Arthur Neiva building was thriving, inspiring and challenging: a lot of young researchers, post-docs, graduate students (M. Sc. and D. Sc. candidates), undergraduates trainees, and Senior researchers were in constant discussions and collaborations in new projects using molecular biology methods to Chagas disease, leishmaniasis, malaria, toxoplasmosis, onchocercosis, giardiasis, echinocososis, schistosomiasis as well as the research on molecular diversity of vectors such as triatomines, simuliids, mosquitos. Most of these subjects were part of the research portfolio of the extinct LEMDI in collaboration with Coura’s research group members. In a space so much crowded it is quite natural for tensions between people to appear frequently. That is why it was so challenging to work out the collaborations within the research groups, despite being a thriving and, to certain extent, creative environment. But at the end of the day all tensions would converge to a single person in this research structure: the leadership and authority of José Rodrigues Coura. Whichever the trouble, the question, the doubt, the stalemate, the demand, the outcry, the hard choice, Prof. Coura would settle the matters and propose a solution, an outcome. Of course, his decision would not always be welcome by some of the people involved and yet what really did matter was that a decision would be made and everyone would respect it. He was the Senior researcher who had founded the department of Tropical Medicine and its graduate program, and nobody else in that place would exhibit the same level of influence and leadership over that bold group of young and middle career researchers. In a certain way this reminds me of a Latin quote acknowledging the power and relevance of ancient Rome: *Roma locusta causa finita*.

The journal Memórias do IOC was created by a federal decree in 1907, and published its first issue in April 1909. Currently MIOC celebrates 113 years of publication in the field of human infectious diseases, an uncommon accomplishment in Brazil, a country of both unstable and limited support to science and technology. From 1976 to 1980, Memórias suffered an interruption in its publication activity, and remained in a “dormant” state. It is not my goal to address here the causes of this interruption, nevertheless I recognise it as an historical event that deeply changed the pathway (and characteristics) of the journal. Once its publishing operation was resumed by Prof. Coura, an entirely new journal was opened to the research community of human infectious diseases.

According to my personal conversations with Prof. Coura about MIOC, soon after he was appointed director of IOC in 1979, he found the Memórias completely abandoned, with no issue published since 1977, a pile of manuscripts waiting for reading and first decision, issues not sent to print, no resources, no adequate equipment. He then set as priority job “to bring Memórias do IOC back to publishing life”, restoring its relevance for the Brazilian Science publishing landscape. That was a true “Redeem Mission”, and it was skillfully done! After restarting the publishing operation in 1980 MIOC left behind its institutional feature (*e.g.*, publishing articles only from IOC researchers) and has become an international journal open to researchers from every location of the world. Prof. Coura also established a fixed editorial board, and formally introduced the peer review (it is not clear to me whether in the years prior to Prof. Coura the manuscripts were sent to external peer review). The journal would keep publishing articles in Portuguese until 1989, when the editor Eloi Garcia decided to accept only articles written in English.

Nowadays MIOC is a global journal complying with the best editorial practices, committed to use its resources to keep pace with technological advances, and embracing the open science. The sequential efforts in the last 42 years that have given Memórias do IOC its current face has one single origin: José Rodrigues Coura, who is certainly a name equaled in importance to the two other iconic persons in Memórias timeline - Oswaldo Cruz (the founder) and Carlos Chagas [the scientist who described the new American trypanosomiasis and published in Memórias his monumental work (1909)].

Prof. Coura took office in 1979 as director of IOC, and simultaneously to the job of redeeming the MIOC, he engaged in a series of reforms to strengthen and renew the then octogenarian IOC. He built an organisational structure based on new departments, invited external scientists to either lead them or establish new laboratories, and created the second graduate program of the Institute. The overall result of these scientific and managerial reforms was the consolidation of the IOC as one the most relevant research organisation in human infectious diseases of Latin America. Prof. Coura’s organisational reform lasted for 27 years. After several rounds of collective discussions starting in 2005, director Tania Araujo Jorge got favorable vote from most of the researchers for the extinction of the research departments and formally assembled in 2007 a new organisation grounded on research laboratories. Prof. Coura vehemently opposed this transformation. It was very hard for him to watch the remodeling of the structure that he has carefully built, and to defend his legacy he took extreme and opinionated positions. For me, personally, it was also a tense moment because I had been sympathetic to the transformations proposed by the new director.

Though we were in opposite sides of our intramural political dispute, I learned invaluable lessons from him during the intense discussions about the new organisation of the Instituto Oswaldo Cruz. At the height of these discussions, Prof. Coura told me: “*you and your colleagues are making a big mistake. A director who has to deal with more than 70 lab heads will not rule properly and will not make up good decisions*”. Only time will tell us whether Prof. Coura was right...

My last politically driven discussion with Prof. Coura was related to the proper utilisation of the heritage building Arthur Neiva, the place of the extinct department of tropical medicine and its experimental facilities. Prof. Coura refused the proposal of reallocating the laboratories to another building in order to afford them a better infrastructure and transforming the Arthur Neiva building in the ‘Teaching Facility of the IOC”. Following a couple of long messages exposing our conflicting views about this subject, which remained a very controversial topic in our conversations from this time on, Prof. Coura interrupted the discussion by quoting Hippocrates: “*life is short, art is long*”.

José Rodrigues Coura was a great researcher, a pragmatic reformer, a tenacious leader, a strong-minded person. He left us important scientific contributions described in hundreds of articles (in Memórias do IOC he published 99 articles), a comprehensive book on human infectious disease, a revitalised and modernised scientific journal (MIOC post 1980), a strengthened and renovated scientific organisation (IOC post 1980), a graduate program in Tropical Medicine, a program to train laboratory technicians, and dozens of D. Sc. and M. Sc. trained and nourished by him for research in human infectious diseases.

He was an example of resolute leadership and willingness to accept the hardest challenges.

Too many accomplishments for a single person in a single life.

*Ars longa*...

